# Suicide prevention psychosocial interventions for youth in low- and middle-income countries: systematic review

**DOI:** 10.1192/bjo.2025.10902

**Published:** 2025-11-14

**Authors:** Mueen Abid, Alishba Iqbal, Warren Mansell, Ayesha Khaliq, Wasima Shehzad, Salman Shahzad

**Affiliations:** Department of Psychology, Air University Islamabad, Islamabad, Pakistan; Department of Clinical Psychology, Curtin enAble Institute, School of Population Health, Curtin University, Perth, Australia; Pakistan Institute of Living and Learning, Karachi, Pakistan; Department of English, Air University Islamabad, Islamabad, Pakistan; Institute of Clinical Psychology, University of Karachi, Karachi, Pakistan

**Keywords:** Evidence-based interventions, mental health, self harm, suicidal ideation, suicide attempt

## Abstract

**Background:**

Suicide is a significant global public health problem, with a disproportionately large burden among youth in low- and middle-income countries (LMICs). Despite growing awareness of the problem, evidence-based interventions in these settings are scarce.

**Aims:**

This systematic review aims to identify and synthesise the evidence-based literature on the effectiveness of psychosocial-interventions to prevent suicide among young people aged 10–24 years in LMICs to reduce the risk of suicide and improve their mental-wellbeing.

**Method:**

After registering protocol with the PROSPERO database of systematic reviews (CRD 420251016364), we searched electronic databases (e.g., PubMed, Medline, Cochrane Library, APA PsycINFO, Scopus, EMBASE, Web of Science and Google Scholar) for potential studies. We considered relevant literature in the English language and published from January 2000 to March 2025. Studies eligible for inclusion were psychosocial interventions compared with a control group, conducted on adolescents in LMICs, and with suicidal-ideation and suicide attempt as primary outcome. Reducing symptoms of anxiety and depression as well as improvements in quality of life were considered as secondary outcomes.

**Results:**

Among 1,223 identified studies, only four met the inclusion criteria. Despite the limited evidence base, all included trials reported reductions in suicidal ideation and improvements in emotional well-being, suggesting the potential effectiveness of culturally adapted psychosocial approaches. Estimated intervention effect sizes ranged from large to extremely large (Cohen’s *d* = 1.46, 2.08, 1.30 and 3.02, respectively), compared with small-to-moderate effect sizes from high-income countries (*d* ≈ 0.24 to 0.54). Secondary benefits were noted for hopelessness, depressive symptoms and quality of life. However, interpretation is limited by small samples and inconsistent methods, reducing comparability with high-income data.

**Conclusions:**

The review highlights major gaps in youth suicide prevention within LMICs, emphasising the urgent need for contextually relevant, evidence-based psychosocial interventions and policy frameworks. Findings suggest moderate effectiveness of current interventions, underscoring the importance of culturally tailored implementation to enhance impact.

Suicide is a major cause of death among adolescents globally, with the majority of burden in low- and middle-income countries (LMICs), where almost 80% of all suicides take place.^1^ The World Mental Health Report of the World Health Organization (WHO) presented suicide as a prominent global public health concern, with approximately 727 000 deaths annually – predominantly in LMICs. It is a leading cause of death among young people between the ages of 15 and 29 years. Men have a higher tendency to die by suicide, but women account for greater attempts and ideation. The majority of suicides are attributed to mental illnesses like depression and alcohol or drug use. For each suicide, there are more than 20 attempts, highlighting the broader impact. Even with some reduction in global suicide rates, progress remains insufficient.^2^ Globally, there are about 1.8 billion young people between the ages of 10 and 24 years – the biggest youth population in history. About 90% of them reside in LMICs. From these estimates, it is estimated that roughly 1.6 billion adolescents and youth aged between 10 and 24 years live in LMICs. This demographic shift highlights a critical public health priority: preventing suicide in young people in settings where mental health services are scarce and the risk of premature death is high.^3^

Adolescence is a critical developmental age marked by increased susceptibility to mental health problems, such as depression, anxiety and suicidal thoughts. The high risk of suicide during this life stage makes it a strategic time for prevention.^4^ Yet, for LMICs, there are usually substantial barriers to the implementation of evidence-based mental healthcare, based on limited infrastructure, shortages of human resources, cultural stigma and competing priorities for physical illness. Consequently, the treatment gap remains wide, and effective, scalable suicide prevention strategies tailored to youth in these settings are urgently required.^5^

During the past two decades, global initiatives like the WHO’s Mental Health Gap Action Programme^6^ and the ‘LIVE LIFE’ suicide prevention strategy have sought to incorporate mental healthcare, including prevention of suicide, into larger health systems, specifically targeting LMICs.^7,8^ In addition, suicide prevention has been integrated into the United Nations Sustainable Development Goals, with the specific goal of decreasing the worldwide suicide rate by a third by 2030.^9^ These efforts place a spotlight on suicide prevention as a worldwide priority, but efforts in LMICs continue to be hampered by a lack of contextually relevant evidence.^10^

Although several systematic reviews have investigated suicide prevention interventions around the world, very few have targeted psychosocial interventions with youth in LMICs. Further, existing reviews have mainly focused on interventions targeting adults or general populations, rather than exclusively on adolescents.^10–12^

To bridge this gap, we performed a systematic review of peer-reviewed articles analysing psychosocial interventions for preventing suicide among young people in LMICs. Our goal was to assess the extent, nature and efficacy of psychosocial interventions that aim to prevent suicidal ideation and suicide attempt in youth aged 10–24 years. This insights for researchers, mental health practitioners and policy makers seeking evidence-based strategies to reduce suicide risk among youth in LMICs, and to highlight key areas where future research and investment are required.

## Method

### Protocol

This systematic review was conducted in line with the Preferred Reporting Items for Systematic reviews and Meta-Analyses guidelines for reporting systematic reviews and meta-analysis,^13^ and registered with the International Prospective Register of Systematic Reviews database of systematic reviews (identifier CRD 420251016364).

### Search strategy

An extensive literature search was conducted by a health sciences librarian in consultation with the research team. Following piloting, refining and completion of a core search plan in Medline, the librarian mapped the accepted strategy by using database-specific subject headings, search fields and operators, for use across the different bibliographic databases: Medline, PubMed, Cochrane Library, APA, PsycINFO, Google Scholar, EMBASE, Scopus and Web of Science. Besides the main databases, we added Google Scholar to cover grey literature and non-indexed studies. This was especially necessary considering the scarcity of evidence on suicide prevention in LMICs, where pertinent theses, reports and smaller-scale studies might not be indexed in main databases. Adding Google Scholar therefore minimised the chances of omitting potentially valuable evidence. The strategy consisted of four key concepts merged with Boolean operators: (a) suicide (e.g. ‘self-harm’, ‘self-injurious behaviour’, ‘suicide prevention)’; (b) psychosocial interventions (e.g. ‘intervention*’, ‘psychotherapy’, ‘psychological therapy’); (c) adolescence (e.g. ‘young people’, ‘teenagers*’, ‘youngsters*’); (d) LMICs (e.g. ‘Nigeria’, ‘Pakistan’, ‘India’). Each concept was searched with database-specific subject headings, natural language keywords and advanced search operators to ensure complete retrieval of relevant studies across databases. Databases were searched between the year 2000 up to the time of search (April 2025). Rayyan 2025 for Windows 11 (Rayyan, Cambridge, Massachusetts, USA; see https://www.rayyan.ai) was used for systematic review screening and data management. The duplicates were removed and the two reviewers (M.A. and A.I.) independently screened the title and abstract. Subsequently, the full-text articles were screened by the same two reviewers to assess for eligibility in this systematic review. At both levels, disagreements were settled by a third reviewer (A.K.). The primary search yielded 1223 studies, but following the elimination of duplicates, 1058 titles and abstracts were screened for eligibility.

### Inclusion criteria

The study population comprised young people aged between 10 and 24 years who were residing in LMICs (as defined by the World Bank). We included psychosocial interventions aimed at suicide prevention, including but not limited to cognitive–behavioural therapy (CBT), dialectical behaviour therapy (DBT), family-based interventions and school-based programmes. Studies were required to have a control group receiving no intervention, treatment as usual (TAU) or other interventions. Study outcomes were reduction in suicidal ideation, suicide attempts, self-harm incidents or completed suicides; and improvement in mental health indicators (e.g. depression, anxiety, quality of life and academic performance). We included randomised controlled trials (RCTs), quasi-experimental studies and cohort studies. All studies were published in English, from 2000 and onward.

### Exclusion criteria

The following studies were excluded: studies focusing solely on high-income countries; interventions not specifically targeting adolescents’ ages between 10 and 24 years; studies with no comparator and studies employing qualitative methodologies, case reports, systematic reviews and meta-analyses.

We only included quantitative and mixed-method studies that described measurable outcomes for suicide prevention. Pure qualitative studies were excluded since the main purpose of this review was to aggregate effect sizes and estimate intervention effectiveness in an equivalent fashion. Although qualitative research can provide rich information on acceptability, cultural appropriateness and implementation processes, such studies lack outcome data that can be statistically pooled.

### Screening and extraction

Two independent reviewers screened abstracts and titles for eligibility (M.A. and A.I.). Potentially eligible studies full-text articles were obtained and evaluated for inclusion in accordance with the eligibility criteria. Disagreements were resolved either by discussion or by referring to a third reviewer (A.K.). Data extraction was as follows: general information, study identifier, title, lead author and contact details, country where the study was carried out, aim of the study, study design, start and end date, study funding sources, potential conflicts of interest for study authors, population description, inclusion criteria, exclusion criteria, method of recruitment, total number of participants, baseline population characteristics, intervention, comparator, main outcome and secondary outcomes.

### Risk-of-bias assessment

The methodological quality of included study was assessed and graded by two independent researchers (M.A. and A.I.). If there were any disagreements, these were resolved by discussion, and a third reviewer was consulted (A.K.). The quality assessment was performed with the Cochrane risk of bias tool. The Cochrane risk of bias tool evaluate the potential sources of bias in RCTs. By taking into account all the criteria, the general quality of the study was given a general ‘good’, ‘fair’ or ‘poor’ grade. Aside from overall study-level judgements, we considered and reported domain-specific ratings such as random sequence generation, allocation concealment, blinding of participants and personnel, blinding of outcome assessment, incomplete outcome data and selective reporting. These disagreements were resolved through discussion, and agreement in all cases was achieved.

### Data synthesis and analysis

The descriptive statistics of articles and respondents’ characteristics were explored. A narrative summary describes the characteristics and findings of included studies. Only four studies that meet the eligibility criteria were finally included in this systematic review. Because of an insufficient number of studies, heterogeneity meta-analysis was not conducted.

### Effect size calculation

When effect sizes were not reported in a standard way in original studies, we calculated standardised mean differences (Hedges’ *g*) for continuous data and odds ratios for dichotomous data. When only test statistics (*t*, *F*, *χ*^2^) or *P*-values were available, we used known conversion formulas to obtain similar effect sizes. When sufficient data were unavailable, authors of the studies were approached. Where further data were not available, these studies were narratively synthesised, but not included within pooled analyses.

## Results

Although the original plan was to perform a meta-analysis, this proved to be impossible because of methodological and reporting restrictions between included studies. The four studies differed significantly in intervention types (e.g. culturally adapted problem-solving therapy, art and music therapy), outcome measures (e.g. the Suicide Ideation Questionnaire – Junior (SIQ-JR), Columbia Suicide Severity Rating Scale (C-SSRS) and Beck Scale for Suicide Ideation (BSSI)) and study designs (RCTs, quasi-experimental and mixed methods). In addition, the absence of homogeneity prevented the creation of a valid effect estimate. Thus, the results were synthesised narratively, and the findings are reported descriptively to emphasise trends and contributions from individual studies without overestimating across different studies with varying methodologies.

### Study characteristics

**Fig. 1:**
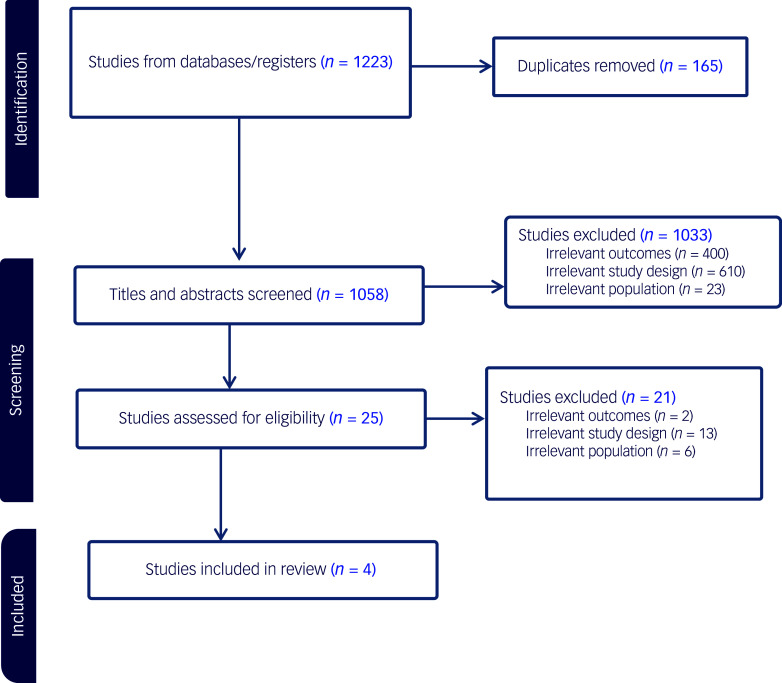
Preferred Reporting Items for Systematic Reviews and Meta-Analyses (PRISMA) chart for record search and screening.

On the basis of previously set eligibility criteria, 25 articles (*n* = 25) were identified for full-text screening. Out of these, four studies (*n* = 4) were included in the final review based on inclusion criteria. The other 21 (*n* = 21) articles were excluded because of ineligibility according to study design, target population, lack of psychosocial intervention, irrelevant outcomes or non-English language of publication (Fig. 1).

Finally, the review included four studies (*n* = 4) from 2014 to 2024, all of which assessed the efficacy of psychosocial interventions for suicidal behaviour in youth with a control or comparator condition. The features and outcomes of these studies are summarised in Table 1.

**Table 1:** Study characteristics and Intervention effects on suicidal ideation, suicide attempts and self-harm

Author, year, country	Study design	Sample	Age±s.d., years	% Male	Intervention (duration)	Comparator group	Measure	Primary study aim	Additional measures	Outcomes	Effect size (Cohen’s *d*)
Husain et al (2014), Pakistan	RCT	Number: 221 history of suicide attempt Intervention group: 108 Control group: 113	Intervention group: 23.2 ± 5.8 Control group: 23.1 ± 5.3	24	Brief psychological intervention – C-MAP (6 months)	TAU	BSSI	Efficacy of intervention, reduction in suicidal ideation	BDI, EQ-5D	Statistically significant improvement on: 1: BSSI (baseline: 21.3 (7.5), 3 months: 7.1 (9.8), 6 months: 7.8 (10.7) 2: Beck Hopelessness Inventory (baseline: 14.5 (5.7), 3 months: 7.9 (8.7), 6 months: 7.5 (8.8) 3: BDI (baseline: 27.8 (15.4), 3 months: 13.0 (16.2), 6 months: 14.8 (17.3) 4: Quality of life (baseline: 39.2 (10.0), 3 months: 55.1 (13.1), 6 months: 63.4 (19.0)	(Cohen’s *d = 1.46*)
Liu et al (2023), Nigeria	Quasi-experimental	Number: 470 female school children who had experienced abduction Intervention group: 235 Control group: 235	Intervention group: 10–27 Control group: 10–27	Intervention group: 0 Control group: 0	Art therapy and music therapy (3 months)	TAU	SIQ-JR	Effectiveness of interventions and reduction in suicidal ideation		Significant reduction in suicidal ideation over time was observed in the response of intervention implementation; *F* (2, 429) 231.171, *P* = .001	(Cohen’s *d* = 3.02)
Alvi et al (2022), Pakistan	RCT	Number: 221 participants with a history of suicide attempt Intervention group: 108 Control group: 113	23.1 ± 5.5	24	C-MAP brief psychological intervention for self-harm (6 months)	TAU	BSSI	Effectiveness of intervention and sustained reduction in suicidal ideation	Beck Hopelessness Inventory	Sustained reduction was observed in suicidal ideation (baseline: 0.05 (0.41), 3 months: 0.66 (0.36), 6 months: 0.63 (0.48)	(Cohen’s *d* = 1.30)
Aggarwal et al (2024), India	Mixed-method approach	Number: 16 participants with a history of self-harm Intervention group: 10 Control group: 6	Intervention group: 14–24 Control group: 14–24	25	ATMAN Intervention (2 months)	TAU	C-SSRS, BSSI	Effectiveness of therapy, reduction in suicidal ideation	GAF, PHQ-9	There were significant differences in the pre- and post-therapy scores on the BSSI (mean difference 16.8 (95% CI 20.2–13.3) and PHQ-9 (mean difference 10.8 (95% CI 14.5–7.04)	(Cohen’s *d* = 2.08)

RCT, randomised controlled trial; C-MAP, Culturally adapted manual-assisted problem-solving therapy; TAU, treatment as usual; BSSI, Beck Scale for Suicidal Ideation; BDI, Beck Depression Inventory; EQ-5D, EuroQoL; SIQ-JR, Suicidal Ideation Questionnaire - Junior; C-SSRS, Columbia Suicide Severity Rating Scale; GAF, Global Assessment of Functioning; PHQ-9, Patient Health Questionnaire-9.

With regard to study design, two studies (*n* = 2) were RCTs,^14,15^ one (*n* = 1) was a quasi-experimental study^16^ and one (*n* = 1) utilised a mixed-method approach.^17^ Geographically, two studies (*n* = 2) were from Pakistan, one (*n* = 1) was from India and one (*n* = 1) was from Nigeria, indicating a representation of South Asia and Sub-Saharan Africa.

A range of assessment tools were utilised to assess suicidal ideation and outcomes. The BSSI was the most commonly applied instrument, used in three of the four studies (*n* = 3). In addition to this, the studies applied a variety of standardised measures to assess general functional and psychological outcomes, including the Global Assessment of Functioning (GAF) (*n* = 1), Patient Health Questionnaire-9 (PHQ-9) (*n* = 1), EuroQoL (EQ-5D) (*n* = 1), Beck Depression Inventory (BDI) (*n* = 1). These measures yielded extensive information regarding emotional, social and occupational functioning among vulnerable youth populations. Overall, the results indicate a positive effect of psychosocial interventions on reducing the suicidal ideation and improved psychosocial functioning in adolescents in LMICs, although the limited number and heterogeneity of studies warrant caution in generalising conclusions.

### Psychosocial interventions for suicide prevention

This systematic review encompassed four studies covering various psychosocial interventions, settings and cultural environments.

Husain et al held an RCT in Pakistan involving 221 individuals (*n* = 221) who had formerly attempted suicide. The intervention group was provided with a brief psychological intervention – a culturally adapted, manual-assisted problem-solving training programme (C-MAP) – over a period of 3 months. Compared with TAU, the intervention group participants showed statistically significant suicidal ideation decreases as assessed by the BSSI, and improved scores on the BDI and EQ-5D. These results suggest that problem-focused, culturally adapted interventions can efficiently alleviate suicidal ideation and underlying emotional distress.^14^

Liu et al investigated the impact of art therapy and music therapy on suicidal ideation in 470 Nigerian female (*n* = 470) youth aged 10–27 who had been abducted. The quasi-experimental study revealed that those in the intervention condition experienced significant decreases in suicidal ideation, as indicated by the SIQ-JR, over a 3-month duration. The findings indicate that expressive and creative therapeutic modalities have potential, non-stigmatising entry points for suicide prevention in trauma-exposed youth in LMICs.^16^

In another RCT, Alvi et al assessed an assisted brief psychological intervention for self-injurious behaviours in 221 people with a previous suicide attempt in Pakistan (*n* = 221). With a 6-month period of intervention, there were significant and prolonged suicidal ideation reductions in the intervention relative to TAU, as evaluated by the BSSI and Beck Hopelessness Inventory. This trial supports the value of culturally adapted, structured psychosocial interventions in producing long-term gains in mental health in youth.^15^

Aggarwal et al applied the ATMAN intervention, a culturally modified treatment programme for Indian youth (aged 14–24 years) with prior history of self-injury. With a small sample size (*n* = 16) and mixed-method methodology, the intervention was found to have promising effects in minimising suicidal ideation, as rated by the C-SSRS and BSSI. The overall psychological functioning according to the GAF and PHQ-9 also improved in all studies, indicating the multi-dimensionality of the intervention’s effect.^17^

There were variations in sample size, intervention and outcome measures, yet all studies showed positive intervention effects on suicidal ideation in children and adolescents in various LMIC settings. Similarities are that both make use of culturally tailored strategies and emphasise individual functioning and emotional regulation. Despite the limited number of studies and heterogeneity of methods, collectively the findings highlight the promise of contextually applicable psychosocial interventions for suicide prevention among youth in low-resource settings.

Standardised effect sizes (Cohen’s *d*) were calculated where possible. Husain et al reported a very large effect (*d* = 1.46) for BSSI reduction over 6 months.^14^ Liu et al demonstrated an extremely large effect (*d* ≈ 2.08) for suicidal ideation reduction following art and music therapy.^16^ Alvi et al observed a large effect (*d* = 1.30) for sustained reduction in suicidal ideation at 6 months.^15^ Aggarwal et al reported an extremely large effect (*d* = 3.02) for BSSI score reduction following the ATMAN intervention.^17^

It is interesting to note that each of the four studies included in this review only examined suicidal ideation as the main outcome, with none assessing suicide attempts, harmful behaviour over time or completed suicide. Suicidal ideation, although an accepted precursor to more violent behaviours and an important target for early intervention, does not always get converted into action. The lack of suicide attempt data in these studies probably stems from the practical and ethical limitations of long-term and large-scale trials in low-resource settings. Nonetheless, this limitation must be considered when determining intervention effectiveness, and future research should work toward including longer follow-up periods and clinically relevant outcomes such as suicide attempts and hospital admissions.

### Quality assessment

The quality assessment of the studies included in our systematic review was carried out with the Cochrane Risk of Bias Tool (RoB 2) (https://www.riskofbias.info/welcome/rob-2-0-tool). The ‘high’ quality studies generally adhere to high methodological standards, showing good randomisation, blinding and outcome reporting; the ‘low’ quality studies exhibit significant methodological flaws, particularly related to high drop-out rates, poor adherence to protocols, and inadequate blinding or randomisation. In our review, each study was ultimately assigned an overall quality rating of good (*k* = 2) or fair (*k* = 2), reflecting its methodological rigour and reliability (Table 2). A domain-specific analysis reported that the vast majority of studies were at low risk of bias for sequence generation and allocation concealment, whereas blinding of participants and staff was commonly rated as high or unclear because of the type of psychosocial interventions. Blinding of outcome assessors was more standardly implemented, but a number of studies evidenced risk of attrition bias as a result of missing outcome data. Selective reporting was overall low risk, although in a minority of instances, the reporting was not detailed enough.

**Table 2:** Quality assessment of included studies

Study number	Author (year), country	Study design	Clinical population	Comparator group	Sample size	Quality rating (good, fair or poor)
1	Husain et al (2014), Pakistan	RCT	People with a history of suicidal behaviour	TAU	221	Good
2	Liu et al (2023), Nigeria	Quasi-Experimental	Female children at risk of suicide	TAU	47	Fair
3	Alvi et al (2022), Pakistan	RCT	People with a history of suicidal behaviour	TAU	221	Good
4	Aggarwal et al (2024), India	Mixed-method approach	Individuals with a history of self-harm	TAU	16	Fair

RCT, randomised controlled trial; TAU, treatment as usual.

## Discussion

This systematic review synthesised evidence on psychosocial interventions aimed at reducing suicidal ideation and self-harm among adolescents in LMICs. Suicide remains one of the leading causes of death among adolescents globally, with disproportionately high rates in LMICs.^7^ Despite this burden, the number of rigorously evaluated interventions developed and tested in LMICs remains very limited. This review identified only four studies – two RCTs, one quasi-experimental study and one mixed-methods trial – conducted in Pakistan, India and Nigeria. Across these studies, intervention effects on suicidal ideation were consistently large to extremely large, with Cohen’s *d* ranging from 1.30 to 3.02. These values are markedly higher than those typically reported in high-income countries, where meta-analyses of psychosocial suicide prevention strategies have found moderate effect sizes for problem-solving therapy (*d* ≈ 0.54),^18,19^ and small-to-moderate effects for digital or creative interventions (standardised mean difference: 0.24–0.45).^20,21^

Several factors may explain these larger effect sizes in LMIC studies. First is greater symptom severity at baseline, as participants often presented with higher suicidal ideation scores at intake, increasing the scope for measurable improvement. Second is the cultural adaptation of interventions, as all interventions were explicitly tailored to local idioms of distress, norms and language, a factor shown to enhance engagement and retention.^22^ Third is that many participants had never received mental health treatment before the trial, potentially heightening responsiveness to structured psychosocial support. Finally, there was intensive provider–participant engagement through frequent follow-up contacts and strong therapeutic rapport, which may have amplified treatment gains compared with less-intensive models in high-income countries.

These factors should be taken into consideration when interpreting the large effect sizes seen in this review. Care should be exercised in comparing these findings with those from high-income nations because a number of contextual factors could be responsible for these variations. Specifically, greater baseline symptom severity, reduced sample sizes and contextual factors such as fewer services available or stigma in LMICs could result in larger effect sizes. These considerations emphasise the need for replication in broader, more diverse samples, and indicate the necessity of paying attention to local context in interpreting intervention efficacy.

### Intervention-specific findings

The interventions reviewed included C-MAP, art therapy, music therapy and the ATMAN intervention.

For C-MAP, both RCTs^14,15^ were conducted in Pakistan and demonstrated significant reductions in suicidal ideation (measured with the BSSI) and depressive symptoms (measured with the BDI). This aligns with broader evidence supporting CBT-based approaches in reducing suicidal thoughts in youth.^23^ Mechanistically, CBT interventions like C-MAP target maladaptive cognitions, enhance problem-solving skills, teach emotional regulation, promote behavioural activation and include safety planning – processes that collectively reduce suicide risk and foster resilience.^24–26^

For art and music therapy, a quasi-experimental study conducted in Nigeria^16^ found that both modalities significantly reduced suicidal ideation among traumatised adolescent girls, corroborating evidence that creative therapies can enhance mood, reduce distress and facilitate emotional processing.^27^ Music therapy in particular may modulate neurobiological stress pathways, increase reward system activation and improve social connectedness.^28^

For the ATMAN intervention, a study in India^17^ conducted a culturally grounded, psychosocial model integrating problem-solving, emotional regulation and family psychoeducation. The mixed-methods evaluation reported improvements in suicidal ideation (BSSI, C-SSRS) and functional outcomes (GAF, PHQ-9). The intervention’s design reflects best-practice principles in suicide prevention research from high-income countries and LMICs, emphasising context-specific adaptation and holistic care.^29^

The magnitude of effects in LMIC studies contrasts sharply with the smaller gains seen in high-income country trials, particularly for brief or low-intensity interventions. Although this discrepancy may reflect genuine differences in baseline severity, intervention adaptation and service accessibility, it also highlights the importance of culturally tailoring interventions rather than directly importing high-income country models without modification.

### Clinical and policy implications

This review strengthens the case for scaling up culturally adapted psychosocial interventions – particularly C-MAP and ATMAN – within LMIC national suicide prevention strategies. Art and music therapies offer low-cost, accessible options for traumatised youth, especially where verbal psychotherapy may be culturally incongruent.

Scaling these interventions will require the following: task-shifting approaches through training non-specialist health workers to deliver evidence-based care;^30^ multisectoral partnerships, linking schools, primary care, community health and social services to create referral and follow-up systems; risk-stratified prevention via implementing universal, selective and indicated suicide prevention programmes, as outlined in the WHO LIVE LIFE framework; and integration into existing systems by embedding suicide prevention into primary health, maternal health and adolescent services, to maximise reach.

The observed large effect sizes suggest that when interventions are both culturally resonant and accessible, substantial reductions in adolescent suicidal ideation are possible, even in low-resource contexts. Future trials should prioritise methodological rigour, gender balance, longer follow-ups and exploration of cost-effectiveness to guide sustainable implementation.

Aside from intervention effects, it is critical to examine the psychological processes by which suicide prevention interventions might have their effects. There is some evidence to suggest that emotion regulation improvement, hopelessness reduction, problem-solving ability enhancement and increased social connectedness are potential mechanisms underlying suicidal ideation and behaviour reduction. Although these processes were not measured directly within the studies included, such processes should be investigated in future research more directly, to inform the development of targeted and scalable interventions.

### Limitations and suggestions

A key strength of this review is its exclusive focus on LMICs and youth populations, a group often underrepresented in global suicide prevention research. The findings offer valuable initial evidence on the potential of culturally adapted psychosocial interventions in these settings.

Nonetheless, several limitations must be acknowledged. First, the small number of included studies (*n* = 4) restricts the breadth and applicability of conclusions across the diversity of LMIC contexts. Second, considerable heterogeneity was evident in study designs (RCTs, quasi-experimental and mixed-method studies), intervention types (e.g. problem-solving therapy, music therapy, art therapy) and outcome measures, precluding the conduct of a formal meta-analysis. Third, sample sizes were generally small – particularly in Aggarwal et al – which limits statistical power and may lead to overestimation of effects.^17^ Fourth, most trials had short follow-up periods (≤6 months), leaving the sustainability of benefits uncertain. Fifth, interventions predominantly measured suicidal ideation without assessing suicide attempts, self-harm behaviours or functional outcomes, thereby limiting conclusions about clinical impact. Sixth, none of the included studies examined psychological mechanisms of change (e.g. emotion regulation, hopelessness and connectedness) or conducted mediation analyses grounded in theoretical models, reflecting a broader limitation in LMIC suicide prevention research. Seventh, the possibility of publication bias, with the relatively small number of studies included, there is a chance that interventions with null or negative findings are unpublished. This would have added to the larger observed effect sizes and should be taken into account when interpreting our results. Finally, the geographical scope of this review was confined to research from Pakistan, India and Nigeria. This limits generalisability, given that LMIC contexts are highly diverse. Further research is required in underrepresented parts of the world like Latin America, South-East Asia and the Middle East, to establish a wider evidence base for suicide prevention. Further, the short follow-up periods in most included studies restricts our ability to draw conclusions about the long-term sustainability of intervention effects. Longer-term evaluations are needed to assess whether benefits in reducing suicidal ideation and behaviour are maintained over time. The quality of evidence is also weakened by the extremely low sample sizes in some of the included trials, one of which had only 16 participants. Small samples have a higher risk of bias, lower statistical power and can exaggerate intervention effects. These results should thus be interpreted with caution and replicated in larger more powerful trials.

These limitations underscore the need for future research in LMICs that is methodologically rigorous, theoretically informed and culturally adaptable. Intervention development should incorporate co-design methods with youth, caregivers and local practitioners, to ensure acceptability and sustainability. Trials should be guided by established psychological models of suicide risk and resilience^31^ to clarify mechanisms of change and identify intervention components that are universally effective versus those requiring cultural adaptation. Researchers should also explore innovative delivery formats – including digital platforms, mobile applications, and school- or community-based programmes – to maximise reach in low-resource settings. The observation of unusually large effect sizes compared with high-income countries studies is particularly noteworthy and merits further exploration. Finally, long-term, adequately powered studies are needed to assess a broader range of outcomes, including suicide attempts, hospital admission rates and real-world functioning, alongside suicidal ideation. Strengthening research capacity and infrastructure in LMICs is essential to building a robust, globally relevant evidence base for adolescent suicide prevention.

In conclusion, this systematic review highlights the promising potential of culturally adapted psychosocial interventions – such as C-MAP, ATMAN, art therapy and music therapy – to substantially reduce suicidal ideation among adolescents in LMICs. The consistently large to extremely large effect sizes observed in the included studies, which surpass those typically reported in high-income contexts, underscore the importance of locally tailored, contextually relevant mental health strategies. Although the small number of available studies, limited diversity of participants and lack of long-term follow-up temper the generalisability of findings, the evidence points to the feasibility, acceptability and clinical utility of these interventions in low-resource settings.

To strengthen the evidence base, future research should prioritise rigorous RCTs with representative samples, culturally appropriate adaptations, active control groups and extended follow-up periods. Integrating such interventions into existing health, education and community systems – particularly through task-shifting models – offers a cost-effective pathway to address the urgent mental health needs of adolescents at risk of suicide. These approaches, aligned with global frameworks such as the WHO’s LIVE LIFE strategy, have the potential not only to save lives, but also to foster resilience, emotional well-being and social connectedness among vulnerable youth. Subsequent research must also include cost-effectiveness analyses, as findings of both impact and cost will be essential to inform policy and enable sustainable scale-up of suicide prevention interventions across LMICs.

### Future directions and implications

Aside from conventional delivery platforms, our evidence points to the promise of digital interventions – such as mobile health platforms, internet-based counselling and telepsychiatry – to bring suicide prevention further into marginalised settings in resource-scarce contexts. Digital strategies have the ability to overcome stigmatisation, cost and geographical barriers, especially for youth who are comfortable with technology.

Beyond that, there is an increasing demand for South–South partnerships to co-develop, pilot and scale up interventions that are culturally suitable and translatable across LMICs. Through such partnerships, mutual learning, capitalisation on established regional innovations and limited dependence on models from high-income settings alone are possible. This approach not only enhances local leadership, but also supports sustainability and fairness in worldwide suicide prevention interventions.

Subsequent suicide prevention research and interventions in LMICs must also consider intersectional vulnerabilities. Gender-based violence, poverty, displacement and other social inequalities may intersect and cumulatively increase risk for suicidal behaviour and impact intervention effects. Redressing these intersecting determinants through context-specific, equity-oriented strategies will maximise the relevance and effectiveness of prevention efforts.

## Data Availability

Data availability is not applicable to this article as no new data were created or analysed in this study.
